# Dorsal approach with Glissonian approach for laparoscopic right anatomic liver resections

**DOI:** 10.1186/s12876-021-01726-4

**Published:** 2021-03-26

**Authors:** Shaohe Wang, Yang Yue, Wenjie Zhang, Qiaoyu Liu, Beicheng Sun, Xitai Sun, Decai Yu

**Affiliations:** 1 Hepatobiliary and Pancreatic Center & Liver Transplantation Center, The Affiliated Drum Tower Hospital, School of Medicine, Nanjing University, Nanjing, Jiangsu Province People’s Republic of China; 2 Wuxi Medical School, Jiangnan University, Wuxi, Jiangsu Province People’s Republic of China

**Keywords:** Dorsal approach, Hepatectomy, Laparoscopy, Surgical procedure

## Abstract

**Background:**

Laparoscopic anatomic hepatectomy (LAH) has gradually become a routine surgical procedure. However, how to expose the whole hepatic vein and avoid the hepatic vein laceration is still a challenge because of the caudate lobe, particularly in right hepatectomy. We adopted a dorsal approach combined with Glissionian appraoch to perform laparoscopic right anatomic hepatectomy (LRAH).

**Methods:**

Twenty patients who underwent LRAH from January 2017 to November 2018 were retrospectively analysed. Of these patients, seven patients underwent laparoscopic right hemihepatectomy (LRH group), seven patients who underwent laparoscopic right posterior hepatectomy (LRPH group), and six patients who underwent laparoscopic hepatectomy for segment 7 (LS7 group). The paracaval portion of caudate lobe could be transected firstly through dorsal approach and the corresponding major hepatic vein could be exposed from its root to the peripheral branches safely. Due to exposure along the major hepatic vein trunk, the remaining liver parenchyma could be quickly transected from dorsal to cranial side.

**Results:**

The mean age of the patients was 53.8 years and the male: female ratio was 8:12. The median operation time was 306.0 ± 58.2 min and the mean estimated volume of blood loss was 412.5 ± 255.4 mL. The mean duration of postoperative hospital stay was 10.2 days. The mean Pringle maneuver time was 64.8 ± 27.7 min. Five patients received transfusion of 2–4 U of red blood cells. Two patients suffered from transient hepatic dysfunction and one suffered from pleural effusion. None of the patients underwent conversion to an open procedure. The operative duration, volume of the blood loss, Pringle maneuver time, and postoperative hospital stay duration did not differ significantly among the LRH, LRPH, and LS7 groups (*P* > 0.05).

**Conclusions:**

Dorsal approach combined with Glissonian approach for right lobe is feasible and effective in laparoscopic right anatomic liver resections.

**Supplementary Information:**

The online version contains supplementary material available at 10.1186/s12876-021-01726-4.

## Background

Hepatectomy has become a curative procedure for several liver diseases, such as liver neoplasms and hepatolithiasis [1–6]. Since being first successfully performed in 1991 [7], laparoscopic hepatectomy has become a routine procedure [8,9]. However, the technical difficulties and the unique vision of laparoscopy have restricted the performance of laparoscopic anatomic hepatectomy (LAH) remaining in large medical centers [10,11]. The use of an appropriate approach can reduce the operation time and the volume of blood loss, promoting recovery [12,13]. We previously reported the feasibility of LAH using the Glissonian approach combined the major hepatic vein first [14]. However, exposing the whole major hepatic vein is still a challenge because of the caudate lobe through venral approach, particularly in right hepatectomy. Dorsal approach in laparoscopic left hemihepatectomy (LLH) was firstly reported to be efficient in 2014 [15] and allowed surgeons freely to transect the caudate lobe. The key point of dorsal approach was the caudate lobe was first transected by utilizing a caudodorsal magnified view, and the corresponding major hepatic vein could be exposed from its root to the peripheral branches and the liver parenchyma was transected along the major hepatic vein from the dorsal side to ventral side. Therefore, we combined dorsal approach and Glissonian approach in laparoscopic anatomic hepatectomy (LRAH) to quickly transect the caudate lobe and expose the hepatic veins. This surgical procedure is safe and effective for LRAH, including for laparoscopic right hemihepatectomy (LRH), laparoscopic right posterior hepatectomy (LRPH), and laparoscopic hepatectomy for segment 7 (LS7).

## Methods

### Patients

From January 2017 to November 2018, 20 patients underwent LRAH through dorsal approach and Glissionion approach in the department of Hepato-biliary-pancreatic Center and Transplantation Center, the Affiliated Drum Tower Hospital, School of Medicine, Nanjing University. Among the patients, seven underwent LRH (LRH group), seven received LRPH (LRPH group), and six patients underwent LS7 (LS7 group). Seven of the patients had hepatocellular carcinoma (HCC), one had intrahepatic cholangiocarcinoma (ICC), seven had hepatic hemangioma, three had hepatolithiasis (HH), one had hepatic adenoma (HA), and one patient had hepatic angiomyolipoma (HAML). The perioperative indices of all patients are listed in Table 1.

**Table 1:** Patient characteristics

Number	Age range (years)/sex^b^	Diagnosis	Operation time (min)	Pringle maneuver time (min)	Blood loss (ml)	POD (days)	Postoperative complications
Group LRH							
1	50–59/1	HCC	210	60	600	6	None
2	40–49/2	HL	270	30	200	7	None
3^a^	30–39/2	HL	345	45	600	18	None
4	50–59/2	HL	360	40	300	22	Transient hepatic dysfunction
5	30–39/2	HM	320	45	200	8	None
6^a^	60–69/1	HM	340	80	800	9	None
7	60–69/1	ICC	295	45	650	16	Transient hepatic dysfunction
Group LRPH							
8	40–49/1	HA	315	105	300	15	None
9^a^	50–59/1	HCC	335	75	700	9	None
10	50–59/2	HCC	260	45	100	9	None
11	50–59/1	HCC	350	75	500	12	None
12^a^	50–59/1	HCC	375	120	200	16	Pleural effusion
13	50–59/2	HM	220	60	500	5	None
14	50–59/2	HM	250	55	600	7	None
Group LS7							
15	60–69/2	HAML	235	40	200	9	None
16	60–69/1	HCC	260	60	200	6	None
17^a^	60–69/2	HCC	430	130	1000	13	None
18	40–49/2	HM	260	35	200	5	None
19	60–69/2	HM	350	75	200	5	None
20	30–39/2	HM	340	75	200	7	None

POD, postoperative hospital stay duration; M, Male; F, Female; HCC: hepatocellular carcinoma; ICC, intrahepatic cholangiocarcinoma; HL: hepatolithiasis; HM, hepatic hemangioma; HA, hepatic adenoma; HAML, hepatic angiomyolipoma; LRH, laparoscopic right hemihepatectomy; LRPH, laparoscopic right posterior hepatectomy; LS7, laparoscopic segment 7 hepatectomy

^a^Case 3, 6, 9, 12, 17 transfused 2–3 U packed red blood cells

^b^Amending sex from "M" and "F" to "1" and "2", without addressing which sex corresponds to which number

The protocol was approved by the Research Ethics Committee of Drum Tower Hospital. Informed consent was obtained in writing from each patient, and the study protocol conformed to the ethical guidelines of the 1975 Declaration of Helsinki, as reflected by prior approval by the Institutional Review Board.

### Operative procedures

The preoperative evaluation, postoperative management, port arrangement, and positioning of the 20 patients were as described previously [14]. All patients were placed in a left semi-decubitus position. The main surgeon stood on the patient’s left side. The patient was placed in the reverse Trendelenburg position and the central venous pressure was maintained at < 5 cmH_2_O. Five trocars were needed for LAH. One 12 mm paraumbilical trocar and carbon dioxide were used to establish the pneumoperitoneum, the pressure of which was maintained at 10–12 mmHg. A 30° flexible laparoscope was introduced through the paraumbilical trocar, and the other four working trocars were placed surrounding the right lobe. A tourniquet for the Pringle maneuver was set using a Nelaton catheter and vessel tape through a 5 mm incision on the left mid-clavicular line. The Pringle maneuver was performed at 15-min intervals to control hemorrhage. The operation began with the division of the falciform ligament, which exposed the gap between the middle hepatic vein (MHV) and the right hepatic vein (RHV). Next, the gallbladder was resected (in LRH or LRPH). The paracaval portion of the caudate lobe was freed from the inferior vena cava (IVC) by means of the liver hanging maneuver.

First, Glissonian approach served to isolate and dissect the corresponding hepatic pedicles (right pedicle for LRH, right posterior pedicle for LRPH, or the pedicles for S7). Then, the paracaval portion of caudate lobe along the IVC was transected through dorsal approch, and then the demarcation line of the ischemic area appeared on the liver surface. Next, Harmonic shear was used to transect the liver parenchyma between the IVC and main hepatic vein (MHV or RHV) first through dorsal approach from its root to the peripheral branches. The corresponding major hepatic vein exposed from the dorsal approach as the intrahepatic landmark. The liver parenchyma between the diaphragmatic demarcation and the MHV or RHV along the aimed hepatic vein was transected through the ventral approach towards to the root of the RHV. The branches of the hepatic vein were dissected by a Hem-O-lok ligating clip, while the root of the RHV was dissected by an automatic stapler in LRH or LRPH.

**Fig. 1:**
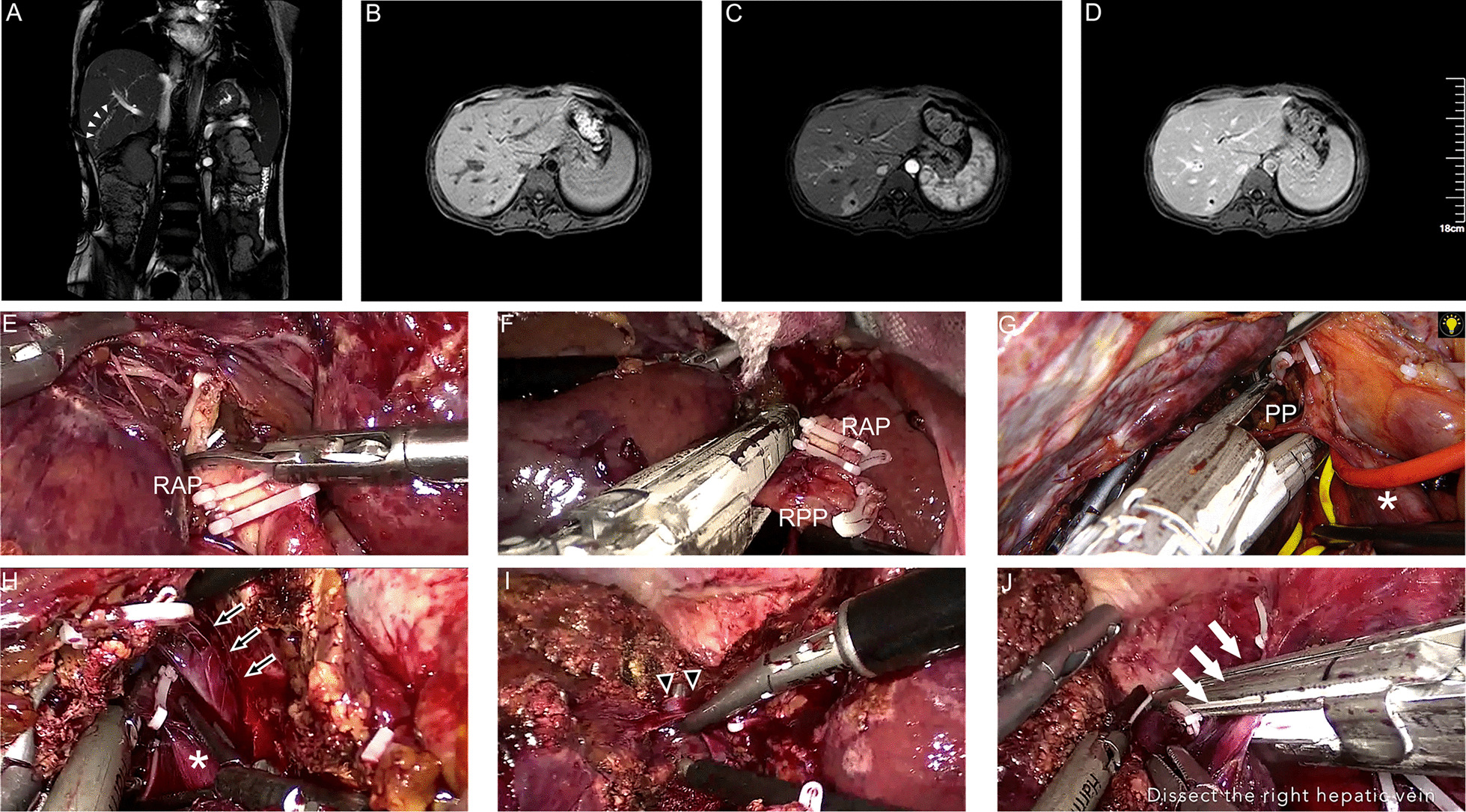
Dorsal approach with Glissonian approach in laparoscopic right hemihepatectomy (LRH). Preoperative MRI showed hepatolithiasis (**a**–**d**) and intraoperative key view during procedure (**e**–**j**): **e** to isolate RAP; **f** to isolate RPP; **g** to transect PP of the caudate lobe between MHV and IVC through dorsal approach; **h** to expose MHV and transect liver parenchymal along MHV through dorsal approach; **i** to isolate S5HV through ventral approach; **j** to dissect RHV. White arrowheads, hepatolithiasis; white asterisk, IVC; black arrows with white edge, MHV; black arrowheads with white edge, S5HV; white arrows, RHV

**Fig. 2:**
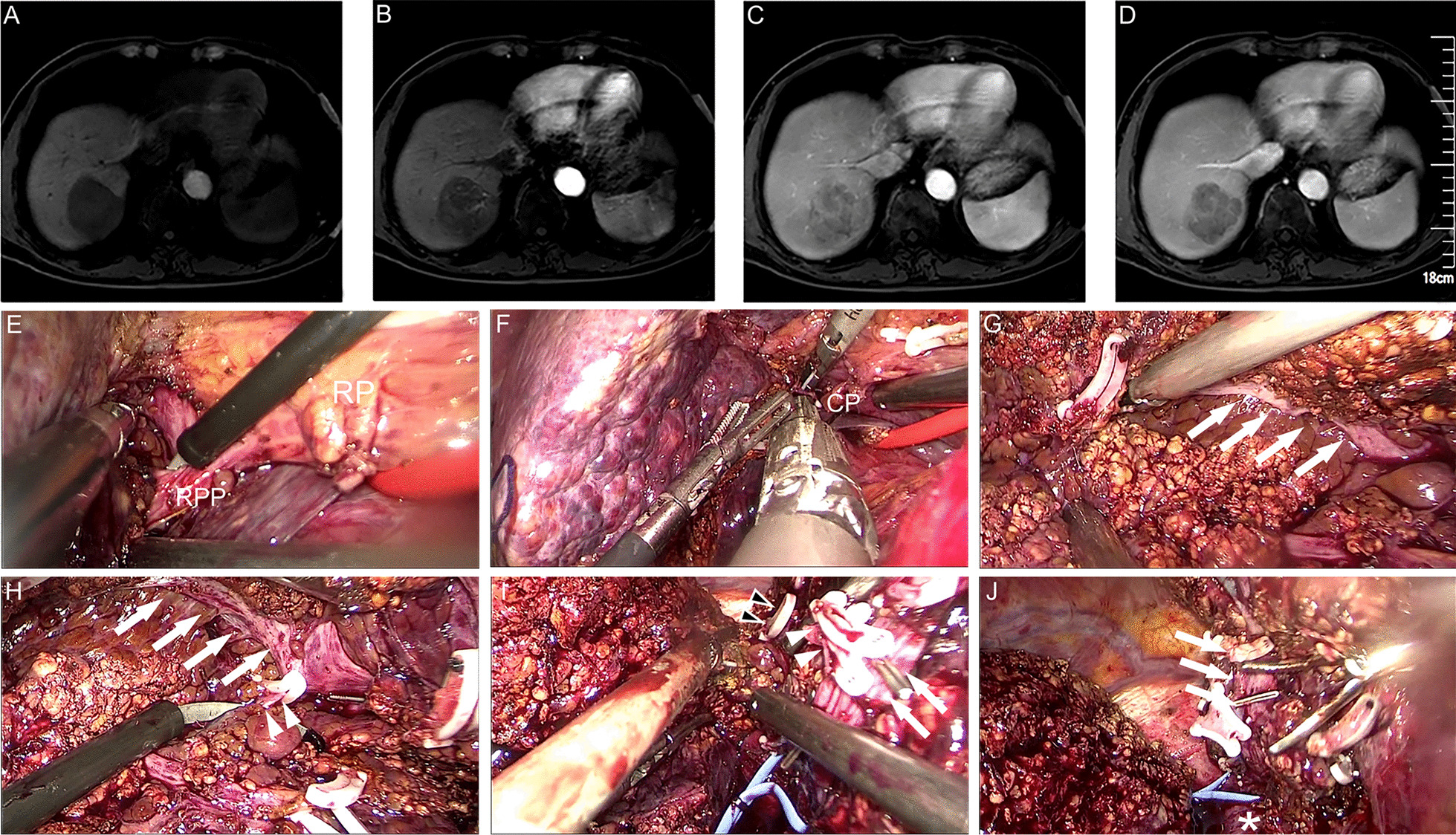
Dorsal approach with Glissonian approach in laparoscopic right posterior hepatectomy (LRPH). Preoperative MRI showed HCC (**a**–**d**) and intraoperative key view during procedure (**e**–**j**): **e** to isolate RPP; **f** to transect liver parenchymal of CP through dorsal approach; G, to transect liver parenchymal between RHV and IVC; **h** to isolate S6HV; **i** to isolate S7HV; **j** the right posterior of liver was transected, and RHV was clearly shown. White arrows, RHV; White arrowheads, S6HV; black arrowheads with white edge, S7HV; white asterisk, IVC

**Fig. 3:**
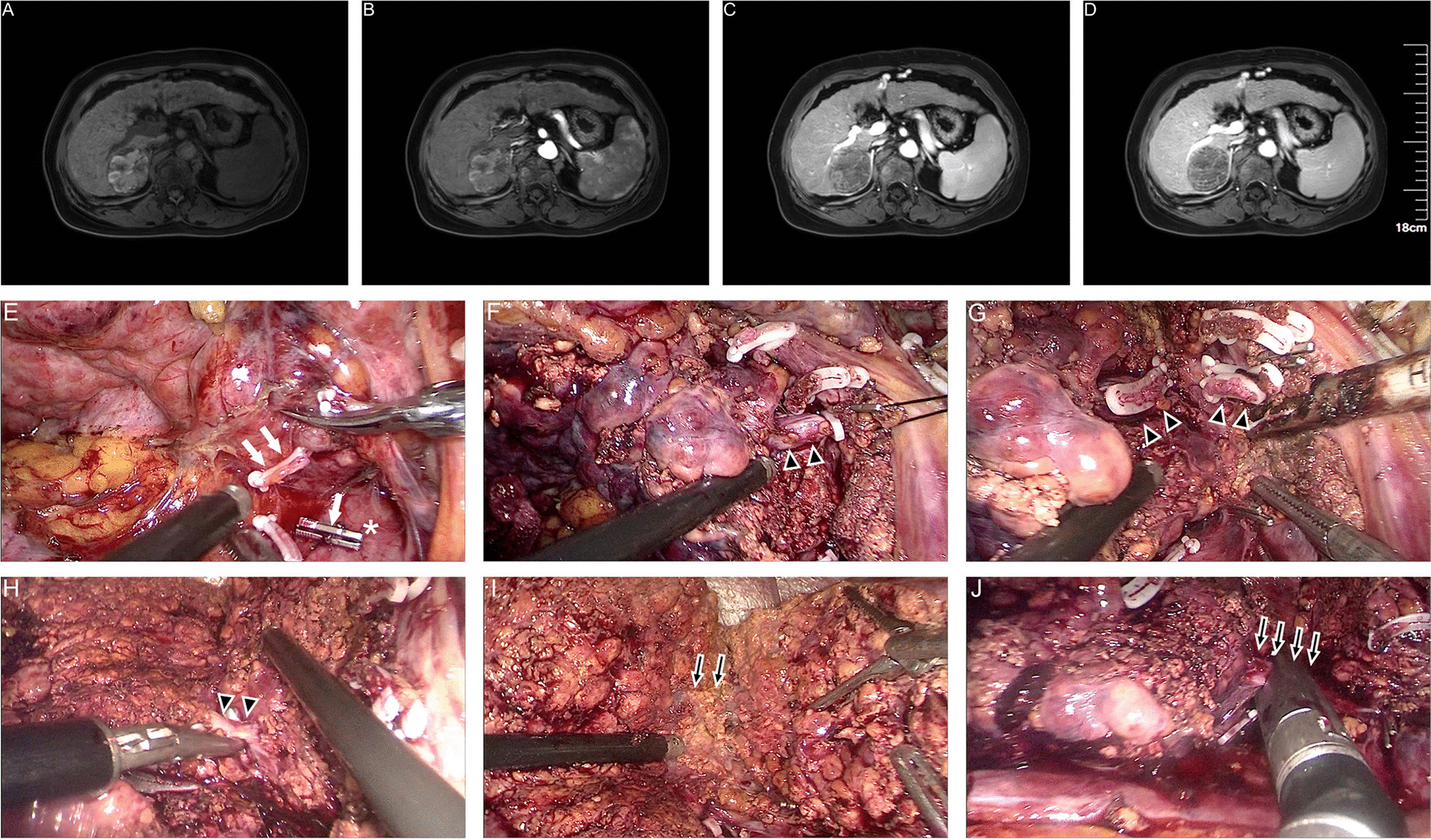
Dorsal approach with Glissonian approach in laparoscopic hepatectomy for segment 7 (LS7). Preoperative MRI showed HCC (**a**–**d**) and intraoperative key view during procedure (**e**–**j**): **e** to isolate the short hepatic vein; **f** to isolate S7P; **g** to transect liver parenchymal through dorsal approach; **h** to isolate S7P through ventral approach; **i** to isolate S7HV; J, the S7HV and S7 was transected. White arrows, the short hepatic vein; white asterisk, IVC; black arrowheads with white edge, S7P; black arrows with white edge, S7HV

Last, the specimen was freed from the coronary, right triangular ligaments, and right adrenal gland. The key procedures are summarized in Fig. 1 and Additional file 1: Video 1 for LRH, in Fig. 2 and Additional file 2: Video 2 for LRPH, and in Fig. 3 and Additional file 3: Video 3 for LS7. The tumor specimen was removed via a mini-laparotomy.

### Statistical analysis

Data analysis was conducted using SPSS Version 21.0 (SPSS, Inc., Chicago, IL, USA). The operative duration, volume of the blood loss, Pringle maneuver time, and postoperative hospital stay duration (POD) were analysed. The data are expressed as medians (ranges) and were compared by one-way analysis of variance or the Kruskal–Wallis test. A value of *P* < 0.05 was considered indicative of statistical significance.

## Results

The mean age of the patients was 53.8 years (range 35–66 years), and the male: female ratio was 8:12. The median operation time was 306.0 ± 58.2 min, and the estimated volume of the blood loss was 412.5 ± 255.4 mL. The mean Pringle maneuver time was 64.8 ± 27.7 min. The mean POD was 10.2 days (range 5–22 days). Five of the patients underwent transfusion of 2–3 U of red blood cells (RBCs). Two patients suffered from transient hepatic dysfunction and one suffered from pleural effusion. The perioperative indices of the patients are listed in Table 1.

In the LRH group (n = 7), the mean operation time was 305.7 ± 52.3 min, and the estimated volume of the blood loss was 478.6 ± 241.3 mL. Patients 3 and 6 underwent transfusion of 2–3 U of packed RBCs. The mean Pringle maneuver time was 49.3 ± 16.2 min. Patient 4 suffered from transient hepatic dysfunction. The mean POD was 12.3 days (range 6–22 days).

In the LRPH group (n = 7), the mean operation time was 300.7 ± 57.8 min, and the estimated volume of the blood loss was 414.3 ± 219.3 mL. The mean Pringle maneuver time was 76.4 ± 27.2 min. Patients 9 and 12 received transfusion of 2–3 U of packed RBCs, and patient 12 suffered from pleural effusion. The mean POD was 10.4 days (range 5–16 days).

In the LS7 group (n = 6), the mean operation time was 312.5 ± 74.1 min, and the estimated volume of the blood loss was 333.3 ± 326.6 mL. The mean Pringle maneuver time was 69.2 ± 34.3 min. Patient 17 underwent transfusion of 4 U of packed RBCs. No patient suffered from serious postoperative complications. The mean POD was 7.5 days (range 5–13 days).

**Fig. 4:**
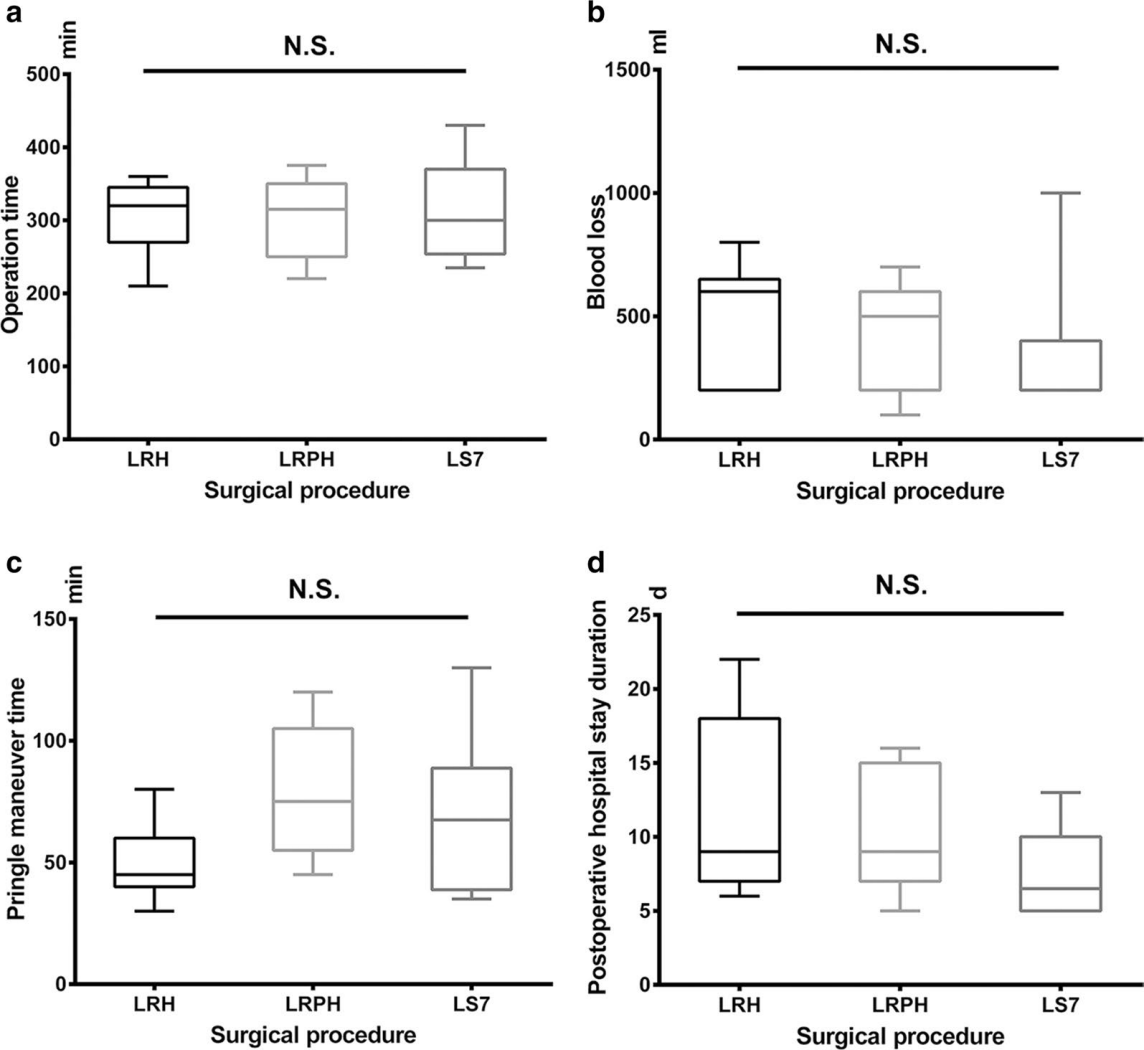
Comparison about operation time (**a**), blood loss (**b**), pringle maneuver time (**c**) and postoperative hospital stay duration (**d**) among Group LRH, LRPH and LS7. There was no significance about operation time (**a**), blood loss (**b**), pringle maneuver time (**c**) and postoperative hospital stay duration (**d**) among Group LRH, LRPH and LS7

None of the 20 patients underwent conversion to an open procedure. The operative duration, volume of the blood loss, Pringle maneuver time, and POD did not differ significantly among groups LRH, LRPH, and LS7 (*P* > 0.05) (Fig. 4).

## Discussion

Anatomic hepatectomy is beneficial for patients with HCC in terms of the recurrence-free survival rate compared with non-anatomic hepatectomy in open surgery, although it may increase the operation time [16–20]. Because anatomic hepatectomy is based on the inflow and outflow corresponding to the target lobe or segment, the root of the Glisson pedicle and hepatic vein can serve as an extrahepatic landmark, while the major hepatic vein can serve as an intrahepatic boundary. Therefore, the pedicle is isolated through Glissonian approach, while the intrahepatic main hepatic vein is located with the guidance under intraoperative ultrasound. It is still a challenge for performers to locate the intrahepatic major hepatic vein under laparoscopy because of the double transformation of two dimensions, especially in LAH, with a long learning curve of close to 50 cases [21,22].

Although the global experience with laparoscopic major hepatectomy is increasing, it is still a technically demanding procedure, especially related to the management of hepatic veins. We reported in 2017 that the pedicles are close to the corresponding main hepatic veins and we could expose the major hepatic vein first through anterior approach [14]. We used this strategy to perform hepatectomies in more than 50 patients. During the operation, we found the caudate lobe hindered us to expose the MHV in LRH or the exposure of the RHV in LRPH. Thus, it is still difficult to expose the hepatic vein without transecting the caudate lobe. An anterior approach is frequently used, which the liver major hepatic vein was exposed from peripheral branches toward the main root, but does not provide the safest exposure to the hepatic veins and inferior vena cava [23,24]. Dorsal approach is an alternative option to dissect the vasculature through a better and safer exposure. Nevertheless, the experience with this approach is scant in the literature [15,^ 25^–27]. This paper describes the technical details of this approach and provides perioperative outcomes from our initial experience.

When performing segmentectomy for S7, dorsal approach cannot be applied to the liver with a thick inferior right hepatic vein. Okuda et al*.* [25] reported six patients underwent LS7 through intrahepatic Glissonian approach with dorsal approach by intercostal trocars, which could increase the risk of intercostal artery hemorrhage and need two more trocars. In our center, the main surgeon stood on the patient’s left side, which could follow an oblique angle and expose the RHV. By means of intraoperative ultrasound, S7 pedicles could be shown from the dorsal vision.

The estimated blood loss is not so low because case 6 suffered from giant hemagioma and case 9, 17 surfferd from severe liver cirrhosis. High quality of randomized controlled trials (RCTs) are needed, and we have registered a Chinese clinical trial in 2018 titled “A randomized controlled trial of Glissonian maneuver combined with dorsal approach and anterior approach: a practical strategy for laparoscopic anatomic hepatectomy” (ChiCTR1800015563). Although it still took us a long time to perform LRAH through dorsal approach and Glissonian approach, the distinct landmark prevented us from “getting lost”.

It is worth noting that when a patient suffered from a huge carcinoma close to caudate lobe, due to little space to reverse the liver and expose the whole IVC, it is difficult to transect the liver through dorsal approach.

## Conclusion

We believe that dorsal approach is a safer alternative to the anterior approach for laparoscopic anatomic liver resections. Our initial experience demonstrates that this approach is feasible. We felt that this approach provided us with a safe exposure to the hepatic vasculature in laparoscopic anatomic right hepatic resections. However, the operation time was approximately 300 min, similar to that of the traditional approach. The sample size was small and it is essential to include more cases for further study.

## Supplementary Information

**Additional file 1: Video 1.** Dorsal approach with Glissonian approach in laparoscopic right hemihepatectomy (LRH)

**Additional file 2: Video 2.** Dorsal approach with Glissonian approach in laparoscopic right posterior hepatectomy (LRPH)

**Additional file 3: Video 3.** Dorsal approach with Glissonian approach in laparoscopic hepatectomy for segment 7 (LS7)

## Data Availability

All data generated or analyzed during this study are included in this published article and its supplementary information files. The datasets generated and analyzed during the current study are available from the corresponding author by email yudecai@nju.edu.cn on reasonable request.

## Abbreviations

LAH: Laparoscopic anatomic hepatectomy
LLH: Laparoscopic left hemihepatectomy
LRH: Laparoscopic right hemihepatectomy
LRAH: Laparoscopic right anatomic hepatectomy
LRPH: Laparoscopic right posterior hepatectomy
LS7: Laparoscopic hepatectomy for segment 7
IVC: Inferior vena cava
MHV: Middle hepatic vein
RHV: Right hepatic vein
RBC: Red blood cell
HCC: Hepatocellular carcinoma
RAP: Right anterior pedicle
RPP: Right posterior pedicle
PP: Paranasal portion
CP: Caudate process
S7P: Glisson pedicles of segment 7
S5HV: Segment 5 hepatic vein
S6HV: Segment 6 hepatic vein
S7HV: Segment 7 hepatic vein
S8HV: Segment 8 hepatic vein
POD: Postoperative hospital stay duration
